# Exploring How People with Expressive Aphasia Interact with and Perceive a Social Robot

**DOI:** 10.1007/s12369-022-00908-8

**Published:** 2022-08-23

**Authors:** Peggy van Minkelen, Emiel Krahmer, Paul Vogt

**Affiliations:** 1 Department of Communication Sciences, Vrije Universiteit Amsterdam, Amsterdam, The Netherlands; 2 Department of Communication and Cognition, Tilburg University, Tilburg, The Netherlands; 3 School of Communication, Media and IT, Hanze University of Applied Sciences, Groningen, The Netherlands

**Keywords:** Human–robot interaction, Socially assistive robots, Chronic expressive aphasia, Complementary speech language therapy, Perception of interaction, Intention to use

## Abstract

People with aphasia need high-intensive language training to significantly improve their language skills, however practical barriers arise. Socially assistive robots have been proposed as a possibility to provide additional language training. However, it is yet unknown how people with aphasia perceive interacting with a social robot, and which factors influence this interaction. The aim of this study was to gain insight in how people with mild to moderate chronic expressive aphasia perceived interacting with the social robot NAO, and to explore what needs and requisites emerged. A total of 11 participants took part in a single online semi-structured interaction, which was analysed using observational analysis, thematic analysis, and post-interaction questionnaire. The findings show that participants overall felt positive towards using the social robot NAO. Moreover, they perceived NAO as enjoyable, useful, and to a lesser extent easy to use. This exploratory study provides a tentative direction for the intention of people with mild to moderate chronic expressive aphasia to use social robots. Design implications and directions for future research are proposed.

## Introduction

Worldwide, 15 million people suffer from a stroke every year, of which a third result in aphasia (www.who.int). Aphasia is a language disorder caused by acquired brain injury mostly as a result of a stroke [1]. Approximately 30–40% of the stroke survivors sustain chronic aphasia, affecting 1.5 million to 2 million people every year. The EU project *Burden of Stroke* shows that between 2017 and 2035 the number of strokes in Europe will increase by 34% and the incidence by 53% [2]. Based on these figures, an increase in the prevalence of chronic aphasia appears likely.

People with aphasia (henceforth PwA) encounter difficulties with spoken language production, understanding spoken language, reading, and / or writing. This impairment of language skills negatively impacts social interactions and limits everyday communication [3]. Extensive research has shown that speech language therapy positively affects PwA’s language comprehension (e.g., listening or reading), and language production (e.g., speaking or writing), compared to PwA who receive no therapy (see [4] for a review). Therapy, however, must comply with a short-term highly intensive dose with a minimum of five to ten hours therapy per week for eight to twelve weeks consecutively to enable significant improvements in language skills [5–7], and an increased use of language skills in everyday life [4]. Short-term high-intensive therapy by speech language therapists proved to be infeasible due to limited funding by health insurers, and the already existing large caseload of aphasia therapists, as Katz et al. (2000) showed in their survey distributed in Australia, Canada, UK, and the USA [8].

Pulvermüller and Berthier [7] suggested the deployment of socially assistive robots (henceforth SAR) in providing additional language training to PwA, i.e., complementary to speech-language therapy. SAR are always available, unlike humans, and therefore might be able to provide the necessary high-intensive language training. Moreover, SAR are, like humans, able to tailor its performance to the user, in this case PwA [9]. Likewise, Pereira et al. [10] formulated a proposal on the deployment of the social robot NAO as mediator in a memory game as part of. Their proposal was positively evaluated by speech-language therapists through a questionnaire based on the Technology Acceptance Model [11, 12]. However, to this date, it is yet unknown if SAR will meet PwA’s needs and requisites in practising language use.

This study, therefore, aims to explore how PwA interact remotely with a SAR that provides a rudimentary training session, how they perceive this interaction, and which factors could influence PwA’s intention to use SAR in practising language. We also conducted one case-study in a live setting in which participant and robot were in the same room. This article is structured as follows: Sect. 2 presents the background of aphasia rehabilitation and reviews related work on SAR. Section 3 describes the semi-structured interaction and questionnaire used in this study as well as the analyses. Section 4 presents the results of the post-interaction questionnaire, thematic analysis, and observational analysis. In Sect. 5, the findings of this study are discussed and suggestions for improvement in robot design for PwA are provided. Finally, Sect. 6 presents the conclusion.

## Background

### Conventional Aphasia Treatment

Traditionally, PwA receive speech-language therapy in a face-to-face setting with a speech-language pathologist. As previously indicated, speech-language therapy for PwA must be provided in a short-term high-intensive dose to significantly improve language skills [5–7]. Breitenstein et al. [6] conducted a randomised controlled trial on 156 German PwA, which yielded a significant effect of high-intensive speech-language therapy of 10 h or more per week for at least three weeks consecutively on verbal communication skills of people with chronic aphasia age 18–70.

Despite this profound evidence, Katz et al. [8] found that PwA rarely receive this amount and duration of treatment, since human therapists simply cannot facilitate this due to lack of time and finances. They conducted a survey on clinicians working with PwA in Australia, Canada, the UK, and the USA, which showed that PwA commonly receive one hour therapy per week. In addition, they found that PwA commonly receive treatment up to one year post stroke, although research has shown no relation between time post stroke and treatment outcome [13]. Similarly, Rose, Ferguson, Power, Togher and Worrall [14] conducted a survey on 188 Australian speech-language therapists, which showed that the current healthcare funding models hamper providing high-intensive speech-language therapy.

### Computer-Based Aphasia Treatment

Due to the lack of possibilities in direct face-to-face treatment, various researchers have started looking for technological alternatives, like computer-based treatment, e.g., [15, 16], or research on tablet-based treatment, e.g., [17–19].

Computer-based treatment enables PwA to increase therapy frequency additional to conventional aphasia treatment [15, 16]. Moreover, Schröder et al. [16] found that specific PwA with mobility problems benefit from computer-assisted aphasia treatment because they are not hindered by mobility problems in travelling to their speech-language therapist.

Like computer-based aphasia treatment, tablet-based treatment enables PwA to practise language additional to their conventional aphasia treatment (e.g., [17–19]). Kurland et al. [19] found that PwA maintained treatment gains from conventional aphasia treatment when using a tablet-based practice program. It is noteworthy, though, that participants required an in-depth training in using a tablet prior to the practice program. Choi et al. [17] investigated an asynchronous tablet-based practice program in Korea which allowed PwA to practise even more often. Similar to Kurland et al. [19], they found that PwA in the USA without practical experience with a tablet required additional training to use the tablet, which negatively affected the aimed self-administered treatment. Kurland et al. [19] researched, similarly like Choi et al. [17], a tablet based self-administered practice program, in which the speech-language therapist met each participant for 30 min per week to monitor their practice frequency. Participants reported feeling bored, and they perceived the sessions as too long. Kurland et al. [19] suggested that the fact that the program was not tailored, negatively affected participants’ engagement.

### Interacting with Socially Assistive robots

A potential alternative, which could address the limitation of screen-based treatments, is the use of social robots. Although not studied with PwA, extensive research has been conducted on interacting with SAR. Like asynchronous screen-based practice programs, robots are—in principle—always available, unlike human therapists, and can be utilised in the home environment of PwA, allowing them to practise language independently, whenever they want. Yet, contrary to screen-based practice programs, SAR were found to have three major advantages in social interaction. These advantages are: (1) physical embodiment, (2) the ability to tailor the interaction, and (3) the ability to use multimodal communication.

#### Physical Embodiment

The first advantage of SAR is its physical embodiment in social interaction that is absent in screen-based systems ([20–25]). Bainbridge et al. [20] showed that physical robots yielded a greater sense of trust compared to onscreen robots, and Björling et al. [21] suggested a moderating effect of the physical embodiment of a robot on stress reduction, compared to an onscreen representation of a robot and a virtual reality robot. Wainer et al. [25], and Okamura et al. [24] found that social robots lead to a higher task engagement and a more pleasant interaction compared to screen-based systems, and Leyzberg et al. [23] found that embodied robot tutors yielded higher learning gains compared to an onscreen representation of the robot. In addition, Breazeal [22] showed that users adhere better to their therapeutic exercises when given by an embodied robot. Broadbent [26] also showed a greater therapeutic involvement to embodied robots compared to screen-based systems. Furthermore, Khosla, Kachouie, Yamada, Yoshihiro, and Yamaguchi [27] demonstrated that social robots lead to a more intuitive interaction by elderly in Australia, which is in line with the findings of Choi, et al. [17], who found that PwA unfamiliar with a tablet assessed the interface as non-intuitive. Winkle, Caleb-Solly, Turton, and Bremner [28] suggested in their focus group study with rehabilitation therapists in the UK, that physical embodiment and the interactive potential of social robots, contrary to screen-based systems, yielded a high user engagement.

#### Personalised and Tailored Interaction

The second advantage of SAR in social interaction is the robot’s ability to personalise therapy to its user, and additionally, use motivational strategies to increase engagement. Van Minkelen et al. [29] researched motivational strategies in the social robot NAO within a second language word learning setting. They found that the social robot needed to fulfil users’ sense of autonomy, provide positive feedback, and relate to the user by personalising the interaction. Similarly, Winkle et al. [28] found that personalization of the interaction is essential for users to maintain engaged with the robot. They proposed that SAR in rehabilitation should personalise and adapt its interaction in real-time, similar to how therapists personalise their therapy to patients, i.e., (1) adapt its style of approach; (2) initiate the interaction; and (3) tailor motivational strategies, engagement and feedback.

#### Multimodal Communication

The third advantage of SAR in social interaction is its ability to use multimodal communication, i.e., they can simultaneously use speech, gestures, and facial expressions. Various research demonstrates that PwA benefit from multimodal communication [30–34]. Eggenberger et al. [30] found an increasement in language comprehension of PwA if their conversation partner used congruent gestures. Since Preisig et al. [33] found that PwA fixate more on gestures than healthy participants, it seems thah deploying a SAR that uses co-speech gestures in addition to speech align with PwA needs. In addition, Matarić, Eriksson, Feil-Seifer, and Winstein [35] found that stroke survivors, although not studied with PwA specifically, enjoyed social robots more, compared to screen-based agents, partly due to its multimodal capacities.

Besides the ability of SAR to use gestures, SAR can recognize gestures made by humans [36]. This capacity is highly relevant for PwA, since various research has found that PwA use gestures to compensate for their verbal limitations [37–40]. Van Nispen et al. [39] demonstrated that approximately a quarter of the gestures made by PwA are essential for understanding their communicative intention. De Wit et al. [36] implemented a gesture recognition algorithm into the social robot NAO to collect a large dataset of naturally made iconic gestures. This dataset, which is publicly available, can be implemented into SAR to enable iconic gesture recognition capabilities.

So, while SAR have the potential to provide PwA additional language training effectively and acceptably, little research has been done to investigate how this could be achieved. We present an exploratory study on how PwA perceive an interaction with a NAO robot with the aim to provide guidelines to develop such robots.

## Method

A mixed method approach was applied by combining a semi-structured interaction and observations (qualitative) with questionnaires (quantitative). This approach allowed for observation and analysis of participants’ interaction with the robot, as well as assessment of participants’ perception of the interaction, to answer the research questions. Due to the Covid-19 pandemic, the interaction took place online, which means that participants had a conversation with the robot via a video call. One participant interacted with the robot in real live as a case study. This study received ethical approval from the Research Ethics and Data Management committee of Tilburg University.

### Participants

A sample of 11 participants (3 female, 8 male) with an age range from 21 to 68 years old (*M* = 52.09, *SD* = 13.61) were recruited using purposive sampling. This entailed contacting all aphasia centres in the Netherlands via email, placing a call on the website http: www.afasienet.com (a platform for PwA, caregivers and practitioners), and by the first author, a former aphasia therapist, approaching PwA personally. Participants were included if they were Dutch native speaking adults with a mild to moderate chronic expressive aphasia who were able to speak intelligibly without any help from anyone. Participants were excluded if they suffered from severe language comprehension problems, psychiatric problems, encountered problems in maintaining attention for thirty minutes, suffered from a progressive disorder, and suffered from severe hearing impairment. All participants received an information letter and a consent form. These two forms contained simplified language to ensure participants understood the content. All participants gave written consent and additional consent on video. Table 1 shows the collected demographics of all participants.

**Table 1:** Demographics of all participants

Participants	Gender	Age	Highest education	Use of AAC during questionnaire
P1	F	63	Secondary vocational	Writing down educational level
P2	M	50	Higher profession	Gesturing
P3	M	60	Secondary vocational	None
P4	M	61	University	Writing down date of birth; Gesturing and making sound
P5	M	21	Higher profession	Writing down date of birth
P6	M	55	Higher profession	Showing image
P7	M	41	Secondary vocational	None
P8	M	68	Secondary vocational	None
P9	M	59	University	None
P10	F	56	Secondary vocational	Typing date of birth and educational level
P11	F	39	Higher profession	None

Participants #1 through #10 interacted online with the robot, so participants and the robot were not in the same room, whereas the researcher and the robot were in the same room. Participants used different platforms for accessing the robot remotely: Skype (*N* = 7), Zoom (*N* = 1), Microsoft Teams (*N* = 1), and Cisco WebEx (*N* = 1). Participant #11 interacted in real live with the robot. The robot, participant, and researcher all sat in the same room.

### Procedure

Participants #1 through #10 (henceforth *'online participants'*) were contacted at an agreed time via a video call using a laptop. The NAO robot and the researcher sat side by side in front of the laptop. The researcher asked participants if they could see and hear her properly, after which instructions were given to improve image and / or sound quality if necessary. The interaction between the robot and participant #11 (henceforth *'live participant'*) took place at the home of this participant.

Each interaction started with the researcher introducing herself, followed by a short introduction about the research, after which participants were asked their consent about the interaction and questionnaire being recorded on video. The *online participants* were then told that the researcher would leave during the conversation. The *live participant* was told that the researcher would distance herself from the conversation physically. The researcher stayed in the same room though to control the robot.

Next, the robot introduced itself by saying '*I am going to introduce myself. Can you hear me properly?*’. At this point the researcher could intervene once more to explain participants how to adjust the audio settings of their laptop. NAO, then, proceeded with: ‘*Hi, I am Robin. It takes some getting used to it for the both of us. Pretty exciting, don’t you think?* Subsequently, the interaction started according to a six-phase procedure (see “Appendix A”). First, the robot explained the setup of the conversation. Second, questions about participants' holidays were asked. Third, the robot announced a change of topic into 'work’ and asked if the participant currently has a job, so questions about 'work' could be asked in the present or past tense. Fourth, participants were asked if they normally use alternative and augmentative communication (AAC) forms when they had not used AAC up until that point of the conversation. The robot then encouraged them to use AAC during the remaining of the conversation. Fifth, questions about participants' work were asked, and finally, the robot ended the conversation with thanking the participant. The number and order of the questions asked about 'holidays' and 'work' depended on participants' responses. For instance, if the participant disclosed in one answer where and with whom he went on holidays with, questions regarding this were passed over. The robot used conversational fillers and clarifying questions to continue the conversation. Importantly, this research focused on how people with aphasia perceived interacting with the robot, therefore, the conversation in itself is the focus of this study and not so much which questions were asked or what answers were given.

After the interaction with the robot ended, the researcher re-appeared besides NAO in front of the laptop camera to introduce the questionnaire to the *online participants*. The *online participants* were asked if they preferred to name the number of their choice, or to make the number clear by raising their fingers. They were also informed that the researcher was going to share her screen so they could see the question and then end the screen sharing, so the researcher appeared full screen. In this full screen mode, the researcher asked them to provide an explanation about their answer. The questionnaire was presented on a tablet to the *live participant*.

After participants finished the questionnaire, they were debriefed about the used WoZ during the interaction, after which the recording was ended.

### Materials

**Fig. 1:**
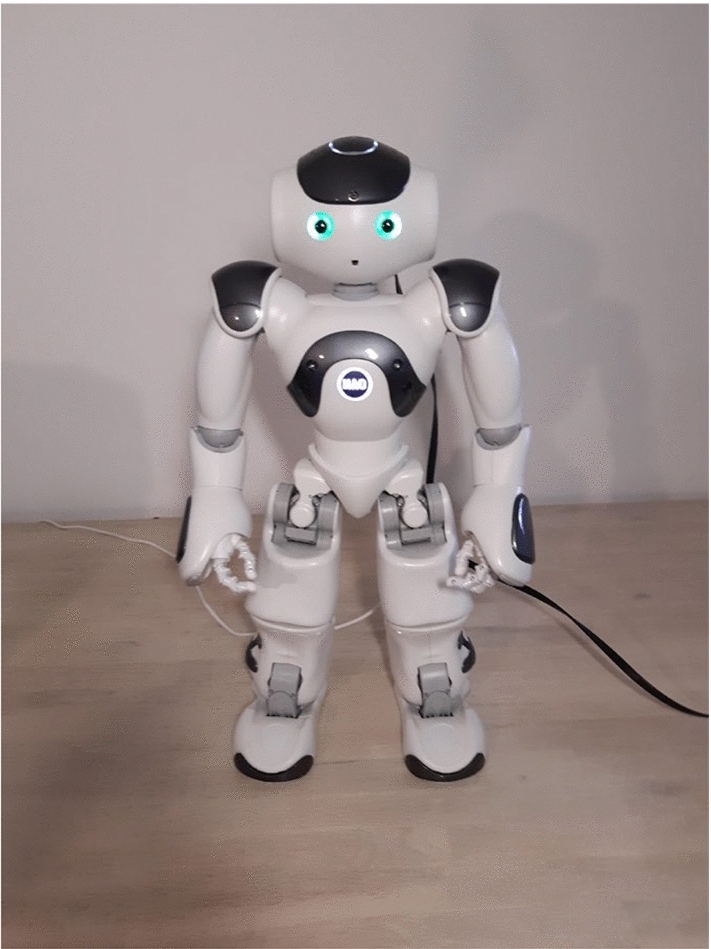
NAO robot

This study used the 58 cm tall humanoid robot NAO (model 25, version 6) with 25 degrees of freedom, produced by Softbank Robotics^1^ (see Fig. 1). The NAO robot was used since it can communicate multimodally by simultaneously using speech and gestures. Eggenberger et al. [30], found that the comprehension of PwA improved when interlocutors use speech and gestures simultaneously. The NAO robot was controlled using Wizard-of-Oz (henceforth WoZ), since the Automatic Speech Recognition of spoken language of PwA is below chance level, and has, to date, not been conducted in Dutch [41]. The WoZ technique allowed for a semi-structured interaction between participants and NAO, when in fact the researcher controlled the NAO robot remotely via pre-programmed robot behaviour, as “Appendix A” shows. The Wizard used in this WoZ paradigm [42] was the researcher who was a former aphasia therapist. This ensured adapted communication to people with expressive aphasia in terms of delayed turn taking during the interaction, so that participants had time to retrieve words, as well as detecting nonverbal communication signals that indicating participants’ retrieval of words, such as withdrawing their gaze from the interlocutor, or in this case, from the camera on the laptop (see [43], pp. 3–4 for on overview of nonverbal signals).

The interaction, which was in Dutch, was designed using Choregraphe version 2.8.6.23 [44], on the NAOqi 2.8 operating system. Co-speech gestures were programmed, since PwA were found to benefit from the additional information provided through co-speech gestures on their language comprehension [30]. These co-speech gestures were non-referential in nature, i.e., they did not hold semantic content, and consisted of arm movements of the robot to accompany concurrent speech. We incorporated pre-programmed gestures using ALAnimationPlayer animations^2^ alongside custom-made gestures for head nodding (as a form of non-verbal backchanneling) and pointing to the camera (deictic gesture) of the laptop were used. This latter gesture was used to indicate the possibility to show alternative and augmentative communication forms in front of the camera. All robot behaviour was triggered using ALDialog user rules and could be triggered independently and in any order. The script of all robot behavoiur is publicly available via the Open Science Framework.^3^ In order to adapt the interaction to language comprehension problems PwA often experience, the robot’s behaviour was set as follows; (i) the speech rate was lowered between 60 and 70% of the default setting, depending on the sentence length; (ii) the pitch was lowered to 90% of the default setting; and (iii) pauses were added to increase comprehensibility, mostly in longer sentences. Conversational fillers were triggered by the researcher to encourage participants to continue talking.

Furthermore, to ensure consistent robot behaviour across participants, autonomous life was switched off, since this setting causes the robot trying to make and maintain eye-contact by moving its head, besides making breathing movements and subtly moving back and forth. The threshold for automatic speed recognition was also adjusted to avoid unintentional triggering of behaviour by unintentionally naming a user rule. A preliminary assessment of the clarity of the interaction as well as the appropriateness of the questionnaire regarding the aimed constructs was performed by two experienced researchers in the field of social robotics. As a result, some minor adjustments were made to the interaction and the questionnaire.

The duration of the interaction was noticeably longer for the *online participants* compared to the *live participant*. For the *online participants*, the duration of the interaction ranged from 6 min and 28 s to 8 min and 57 s, with an average interaction duration of 7 min and 7 s, whereas the interaction duration of the *live participant* was 5 min and 14 s.

To bridge the sound loss for the *online participants*, due to the distance between NAO and the laptop microphone, an external microphone was used which was connected to an Alecto PAS-210 speaker set with a mixer, built-in amplifier, and two speakers. This microphone and speaker set was not used for the *live participant*.

**Fig. 2:**
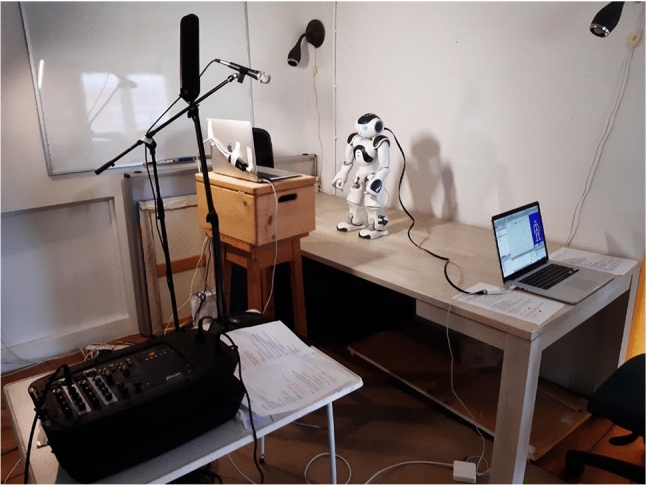
Overview of the study set-up

**Fig. 3:**
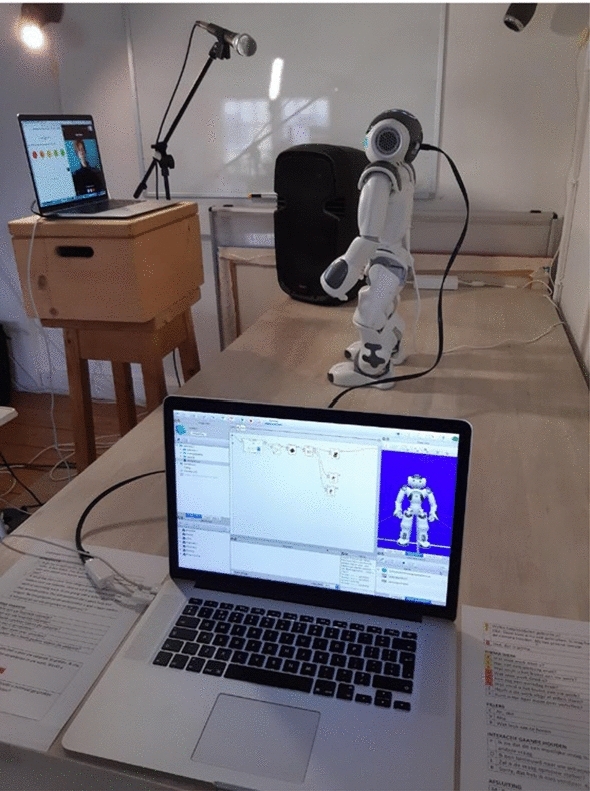
Experimenter’s view of the study set-up. The laptop inthe front was used by the researcher to control NAO. The laptop on the left shows a split-screen with the participant at the right, and the questionnaire at the left

The questionnaire (see 3.2.2) was presented to the *online participants* via a shared screen on the laptop, and on a Microsoft Surface Pro 13-inch laptop with a touchscreen to the *live participant*. Figures 2 and 3 show an overview of the study setup for the *online participants*.

### Measures

To explore how participants interacted and communicated with the NAO robot, participants’ interaction with the robot was manually coded on four general categories that might influence the interaction: (1) simultaneously turn-taking (e.g., participant/ robot starts talking before the turn of the other is completed, or participant and robot start talking at the same time; (2) communication break-down (e.g., internet connection problems) that hindered the interaction; (3) participants’ mood during the interaction; and (4) participants’ involvement in the interaction. “Appendix B” shows the coding scheme with general categories and subcategories. 

To this aim, 2-min video clips were created, starting at question two from the topic ‘holidays’ for one minute, followed by question two from the topic ‘work’ also for the duration of one minute. We started at the second question of both topics (i.e., holidays, and work), so participants could get familiar with the topics and the conversation would be well underway at that point. Clips from both topics were included to reduce possible preferences of participants in terms of the topic.

Frequency counts of the codes 'simultaneously turn-taking' and 'communication breakdown' were collected. Participants' participants' Mood and Involvement during the interaction were coded on a six-point, respectively four-point scale, based on Huisman and Kort [45]. Besides the first author, two experienced raters applied the coding scheme on a subset of four randomly chosen *online participants* (40%), as well as on the single *live participant*. The inter-rater reliability was assessed using a two-way mixed effect model based on the mean-rating (k = 3) and consistency [46, 47], since the design was fully crossed, and the coders were not randomly selected. The intraclass correlation coefficient for inter-rater reliability was 0.93, with a 95% confidence interval of [0.90, 0.95] which can be considered excellent reliability [47].

To assess how participants perceived the interaction with the NAO robot and whether they intend to use the NAO robot, a written questionnaire was administered after the interaction, which the researcher also read aloud. This allowed participants to compensate for spoken or written language comprehension difficulties by focusing on the other language mode.

The post-interaction questionnaire was based on the findings of Heerink, Kröse, Evers, and Wielinga [48], who suggested an adaption of the Unified Theory of Acceptance and Use of Technology (UTAUT) [49] to specifically evaluate the acceptance of social robots. Heerink et al. [48] revealed a significant correlation between Perceived Enjoyment (PE), Perceived Ease of Use (PEOU), Perceived Usefulness (PU), and Attitude (ATT) on Intention to Use. Intention to Use, in turn, significantly predicts the actual Usage of technology. Yang and Yoo [50] stated that the construct Attitude falls apart in two components, namely the cognitive component of attitude and the affective component of attitude. The construct Attitude, as proposed by Heerink et al.[48] focused on the cognitive component of attitude. This component refers to participants’ beliefs regarding the use of the robot. The affective component of attitude, though, refers to how much participants liked the robot. This component was examined through the observational category *Mood* and *Involvement*, based on Huisman and Kort [45]. “Appendix C” shows the model underlying the questionnaire used in this study.

**Fig. 4:**
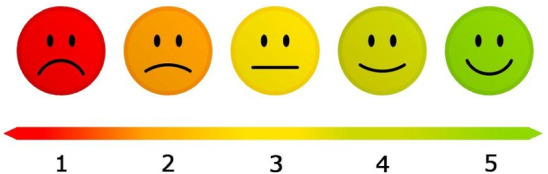
Visual scale used during the questionnaire to enable all participants to rate each question

Each construct, i.e., PE, PEOU, PU, and ATT consists of two questions, which have been translated from English to Dutch and, moreover, adapted to simple, short sentences, without using negative framing, to increase the comprehensibility of PwA. Participants responded to each question on a 5-point Likert scale (1: “not at all”, 5: “very much”), which was supplemented by a visual aid (see Fig. 4). Participants then proceeded to five demographic questions about their age, gender, experience with robots, and education, since Venkatesh et al. [49], found gender, age, and experience with robots to be moderating factors on the Intention to Use. Regarding the moderating effect of education on technology acceptance, inconclusive results were found [51–53]. “Appendix D” shows the questions in Dutch which were used in the questionnaire. Table 2 shows the descriptive statistics of the translated questionnaire items (i.e., translated from Dutch to English) of the four constructs.

**Table 2:** Descriptive statistics of questionnaire items of four constructs and demographics

Questions	Construct	Mean	SD
1. I enjoy the robot talking to me	PE	3.73	1.01
2. I like the robot	PE	4.09	1.22
3. I find the robot easy to talk to	PEOU	3.00	1.00
4. I can hear the robot properly	PEOU	3.73	0.91
5. I think the robot is useful for practising language	PU	3.91	1.22
6. I would like to have more conversations with the robot	PU	3.64	1.21
7. I think the robot can be a good conversation partner	ATT	3.73	1,01
8. I think the robot can assist me practise language more	ATT	3.82	1.33
9. Have you ever participated in a study about a social robot before today?	*NA*	*NA*	*NA*
10. Have you ever interacted with a social robot before today?	*NA*	*NA*	*NA*
11. What best describes your gender?	*NA*	*NA*	*NA*
12. What is your year of birth?	*NA*	52.09	13.61
13, What is the highest degree of education you have completed?	*NA*	*NA*	*NA*

Question 1 to 8 relate to the four constructs. Question 9 to 13 are demographic questions

*PE* Perceived Enjoyment, *PEOU* Perceived Ease of Use, *PU* Perceived Usefulness, *ATT *Attitude, *DE* Demographic questions, *NA* not applicable

IBM SPSS Statistics 25 was used to calculate the Mean and Standard Deviation per question, as well as per construct.

After each question item, participants were invited to explain their answer in more detail to capture their perception of the interaction by answering the question 'Can you indicate why?'. Inductive Thematic Analysis, a bottom-up method to explore the data was used to identify, analyse, and report participants' responses to the open-ended questions. This qualitative analytic method followed the six steps by Braun and Clark [54]; (1) familiarisation with data; (2) generating initial codes; (3) identification of themes; (4) reviewing themes; (5) defining and naming themes; (6) producing a report. The transcripts of participants' responses to the open-ended questions were read thoroughly and analytically using ATLAS.ti 8 ® (Scientific Software Development GmbH), which resulted in the initial coding of categories. Next these codes were sorted into potential themes after which all relevant coded data were systematically collated into the identified themes. Then, potential themes were reviewed and critically assessed on the data that supported these themes, which led to the final themes and subthemes. Representative quotations from participants are used to demonstrate the findings.

## Results

**Fig. 5:**
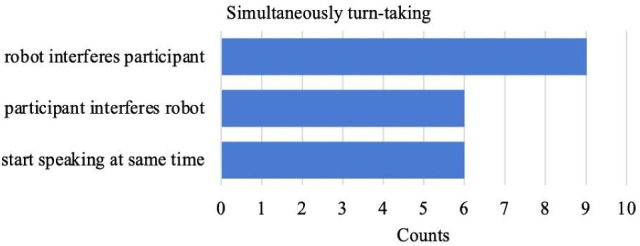
Distribution of frequency counts of *Simultaneously turn-taking* across subcategories

We performed an observation analysis on video recording of the interaction to explore how participants interacted with a NAO robot and to detect factors that might have affected the interaction. The results show that simultaneously turn-taking occurred a fairly large number of times, with the robot interfering the participants more often than vice versa. Interestingly, simultaneously turn-taking occurred with all participants except with the *live participant*. Figure 5 presents the occurrences of simultaneously turn taking across the subcategories 'robot interferes participant', 'participants interferes robot', and 'robot and participant start speaking at the same time'. These findings provide an insight into how turn-taking occurs when PwA interact with NAO within the WoZ-paradigm of this research.

**Fig. 6:**
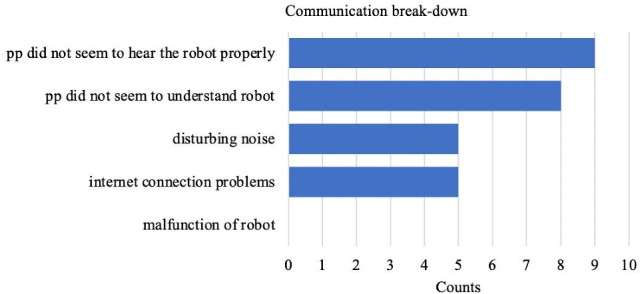
Distribution of frequency counts of *Communication breakdown* across subcategories

The results, furthermore, show nine occurrences of communication break-down where participants did not seem to hear the robot properly. These findings match participants’ perception of the reduced intelligibility of the robot when making gestures. Additionally, eight occurrences of communication break-down were coded due to comprehension problems of participants. Furthermore, five occurrences of communication break-down were coded caused by different sources: somebody talking to the participant during the interaction (2 times); people talking in the background (1 time); notification sound of multimedia device (1 time); and an unidentifiable sound (1 time). Finally, five occurrences of communication break-down were coded due to internet connection problems. Figure 6 shows the frequencies of communication break-down across the categories mentioned above. These findings provide insight into possible reasons why communication between PwA and NAO hampered.

**Fig. 7:**
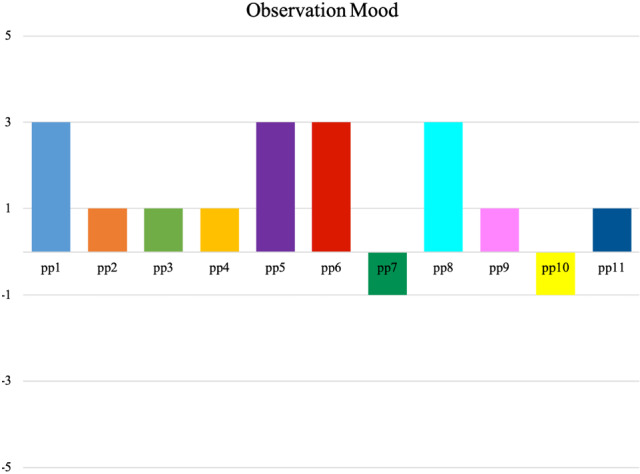
Observation score on *Mood* of two 2-min video clips during the interaction with the robot. Each coloured bar represents one participant. The *Mood* score is given on the y-axis (+ 5: very happy; + 3: happy; + 1: neutral; − 1: small signs of negative mood; − 3: proper signs of negative mood; − 5: very negative mood), based on Huisman and Kort [45]. Participants 1 through 10 interacted online with the robot, while participant 11 interacted live with the robot

**Fig. 8:**
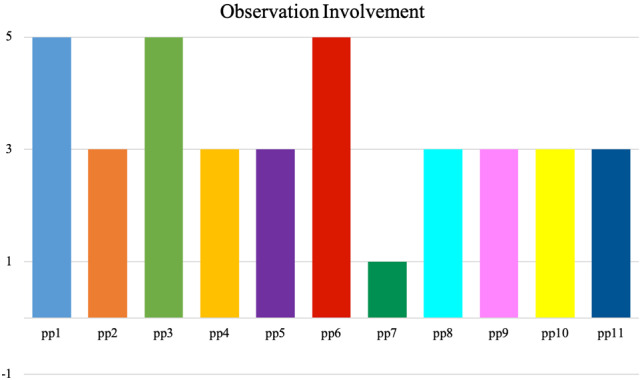
Observation score on *Involvement* of two 2-min video clips during the interaction with the robot. Each coloured bar represents one participant. The *Involvement* score is given on the y-axis (+ 5: very involved; + 3: properly involved; + 1: neutral; − 1: withdrawn), based on Huisman and Kort [45]. Participants 1 through 10 interacted online with the robot, while participant 11 interacted live with the robot

Additionally, participant’s mood and level of involvement were coded. Most participants showed a neutral mood (55%), i.e., they showed no signs of specifically liking or disliking the robot. When examining participants’ level of involvement, the majority (64%) was properly involved in the interaction (see Figs. 7 and 8).

**Fig. 9:**
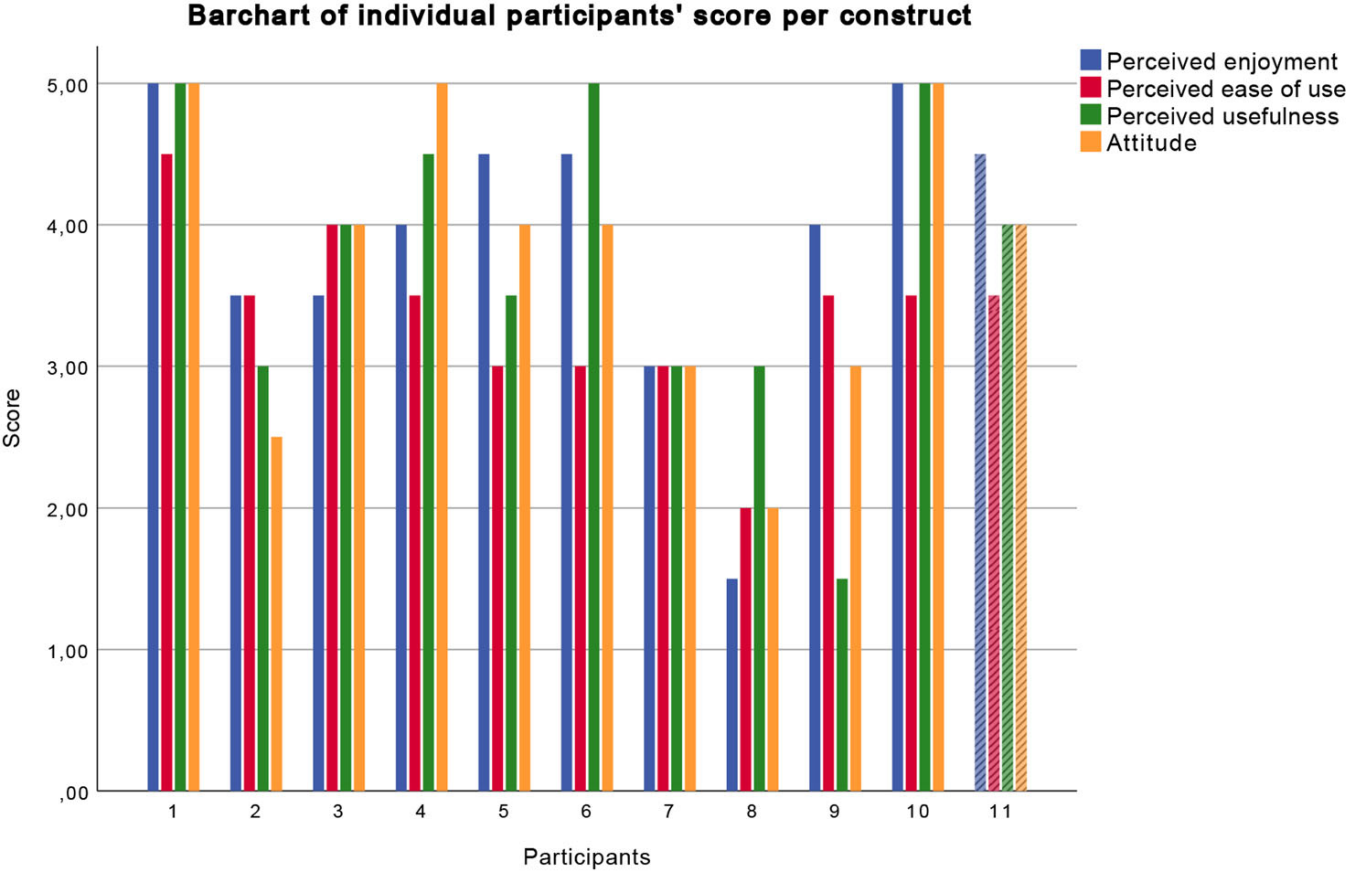
Bar chart showing the ratings per participant per construct. Participants 1 through 10 interacted online with the robot, while participant 11 interacted live with the robot

The results of the questionnaire, showing the four constructs Perceived enjoyment, Perceived ease of use, Perceived usefulness, and Attitude are shown in Fig. 9 per participant. No significant effects of gender, age, and educational level were found. Since none of the participants have ever interacted with a social robot before this research, the factor experience with robots has not been analysed.

**Fig. 10:**
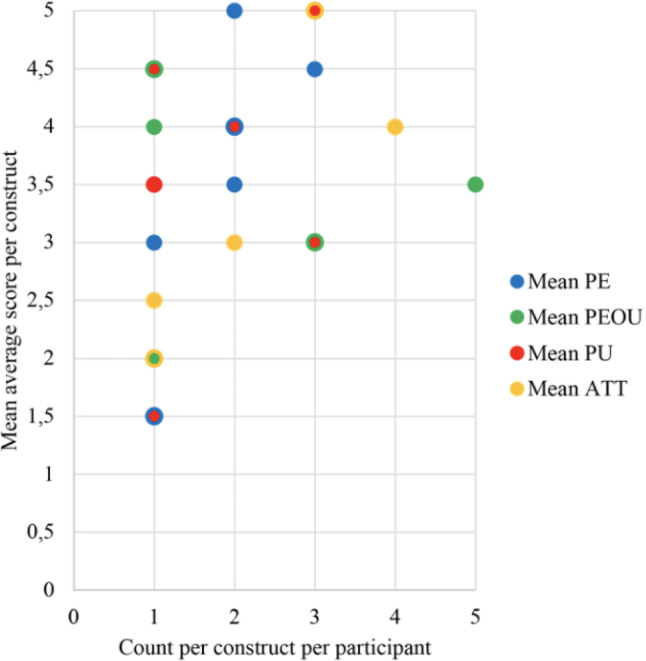
Scatterplot of all participants showing the average score per construct per participant. *PE* Perceived Enjoyment, *PEOU* Perceived Ease Of Use, *PU* Perceived Usefulness, *ATT* Attitude

Next, four one-sample *t*-tests were conducted to determine whether participants' mean scores of Perceived Enjoyment (PE), Perceived Ease of Use (PEOU), Perceived Usefulness (PU), and Attitude (ATT) were different from the middle value of 3.0 on the 5-point Likert scale. Participants' Mean PE (*M* = 3.91, *SD* = 1.02) was significantly higher than 3.0, *M*_*diff*_ = 0.91, 95% CI [0.22, 1.59], *t*(10) = 2.96, *p* = 0.014, *d* = 0.89, indicating that participants enjoyed interacting with NAO above the middle value. Participants' Mean PU (*M* = 3.77, *SD* = 1.10) was also significantly higher than 3.0, *M*_*diff*_ = 0.77, 95% CI [0.03, 1.51], *t*(10) = 2.32, *p* = 0.043, *d* = 0.70, indicating that participants perceived NAO as useful Participants' Mean ATT (*M* = 3.77, *SD* = 1.03) was also significantly higher than 3.0, *M*_*diff*_ = 0.77, 95% CI [0.08, 1.47], *t*(10) = 2.48, *p* = 0.033, *d* = 0.75, indicating that participants believe NAO could be of practical use However, participants' Mean PEOU (*M* = 3.36, *SD* = 0.64) was not significantly higher than 3.0, *M*_*diff*_ = 0.36, 95% CI [− 0.06, 0.79], *t*(10) = 1.90, *p* = 0.087, which means that participants did not perceive NAO as easy to use. Figure 10 shows the scatterplot of the average score per construct (PE, PEOU, PU, and ATT) per participant.

### Thematic Analysis

Three major themes were identified from the transcripts of the open-ended questions, as Table 3 shows: (1) the intelligibility of the robot; (2) participants’ judgments of robot features in interaction; and (3) autonomy in practising language use. Illustrative verbatim quotations of the themes are presented below.

**Table 3:** Themes and subthemes that emerged from the data

Category	Themes	Subthemes
Intelligibility of the robot	Disturbing noise by robot gesturing	Time required to familiarize to sound of robot
Robot features	Robot was nice and easy to talk to	Robot had good speech rate
	Need for more dialogue with robot	Need for more cueing in word retrieval
Autonomy in practising	Need to expand frequency and duration of practising language use	

Themes were mentioned > 50% of participants; Subthemes were mentioned < 50% participants

#### Intelligibility of the Robot

**Fig. 11:**
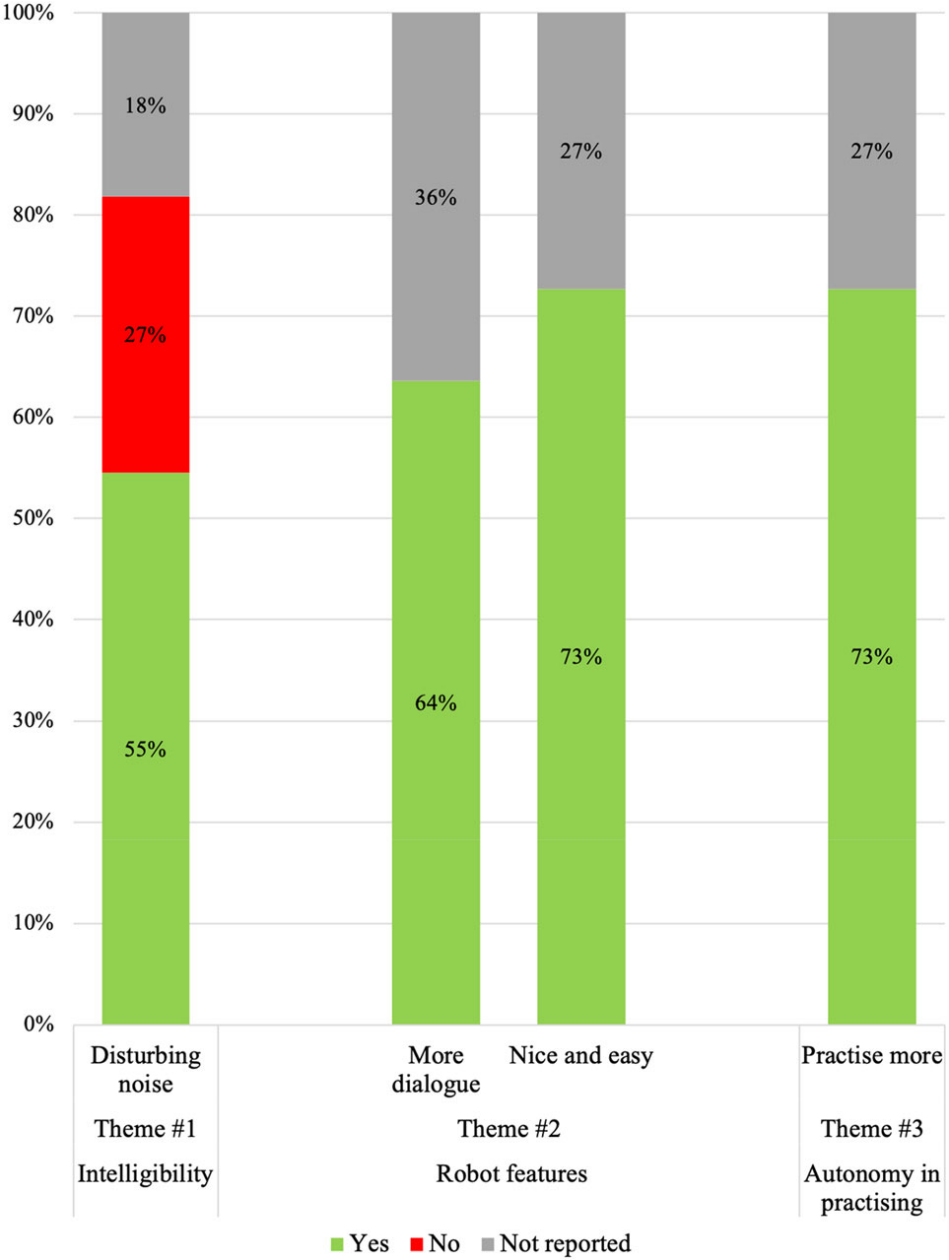
Frequencies of the common themes *Intelligibility*, *Robot features* with two sub-themes, and *Autonomy in practising* that emerged from participants’ responses to the open-ended questions

The first theme that emerged from the open-ended questions was the intelligibility of the robot. Six participants (55%) mentioned that the robot’s gesturing negatively affected its intelligibility, as Fig. 11 shows. They indicated that the moving of arms caused disturbing noise, which made it difficult for them to understand the robot.

Participant 4: *Well, uh then [participant moves arms alternately up and down] also there [participant makes ssss-sound], don’t do that. Just [participant moves arms alternately up and down* + *pp makes rrrr- sound]. Is difficult for me to talk with [participant makes rrrr-sound] so.*

Researcher: *Are you saying that if the robot makes a lot of gestures, it is difficult to understand?*

Participant 4: *Yes, exactly. [The participant moves arms alternately up and down* + *pp makes zzzz-sound]. Researcher: Do you hear him move?*

Participant 4: *For me then uh yes [participant makes a circular movement next to the temple]*.

Participant 2: *Uh, sound, and he does all kinds of things [participant makes big arm movements], and that a little bit, and that uh, that doesn’t go so well than uh*.

Participant 3: *Well, uh the robot that, that talk uh that’s nice but moving the arms of this that thing, it’s almost impossible to follow*.

A subtheme that emerged from the data was that participants (45%) needed to familiarise themselves with the sound of the robot’s speech to understand it properly.

Participant 5: *First, I got a bit difficult. It is a bit better, later it is a bit better.*

Researcher: *Okay, so you had to get used to it?*

Participant 5: *Yes.*

Researcher: *To the sound that the robot made, is that what you mean?*

Participant 5: *Yes, yes*

Researcher: *And then when you get used to it, then ...*

Participant 5: *Yes, that is going better.*

Participant 10: *Uh, wait a while, uh. What did you say? What did you say? What did you say?*

Researcher: *Are you saying that you couldn’t always understand the robot properly?*

Participant 10: *Right, yes.*

#### Participants’ Judgements of Robot Features in Interaction

The second theme that emerged was that participants (73%) highlighted that the robot was nice and was easy to talk to, due to the robot’s slow speech rate and delayed response timing (see Fig. 11). This enabled them to retrieve the right words. They also felt less hesitation to talk, due to the absence of judgement by the robot. They also felt less time pressure in retrieving the right words due to the robot’s neutral attitude.

Participant 11: *Well. Don’t you uh don’t you have say shame.*

Researcher: *So, you mean it’s okay to make a mistake because it’s just a robot?*

Participant 11: *Yes, correct.*

Participant 6: *That robot has no emotion and there you have no embarrassment because then it is safe, eh*, *because then it is safe, eh*.

The third theme that emerged was that participants (64%) indicated that they would have liked more dialogue with the robot, in the sense that the robot would have responded to their answer in a more content wise manner (see figure 11).

Participant 9: *It would be nice if he uh if you can respond to what you say. More uh today’s newspaper or uh whatever, if he that robot can have something unpredictable, can have something predictable. [...] Yes, slightly more variety and is more on random, give it a newspaper headline. What are you doing today? Well, I have to pass an exam today. Then I hope he knows and a somewhat larger repertoire, that he knows what an exam is so to say. Because that I can pick up on that. Or uh I’m going to a party. Nice who’s birthday? Sort of*.

Participant 6: *If uh I had a story and then and then it has a uh conclusion a summary and uh was not the correct description of the story uh uh an example uh holiday. I had, he said, what uh was the nicest or nicest. I wanted the story of uh restaurant I made up completely uh well. But uh, his answer was” oh nice”.*

Researcher: *And what else could the robot have done there?*

Participant 6: *Yes uh, even better interaction.*

Researcher: *So that the response would have been more appropriate?*

Participant 6: *Yes, right yes*.

A second subtheme that emerged from the data was that participants (18%) appreciated that the robot talked slowly, which provided them more time to process language.

Participant 7: *But slowly, slowly.*

Researcher: *The robot talks slowly?*

Participant 7: *Yes, that is good.*

Participant 4: *Uh for me is the slowly. That is good. Too quickly is hard for me. [...] Uh if I uh slowly slowly talking. Also waiting. Not too quickly, but slowly*.

A third subtheme that emerged from the data was that participants indicated that the robot did not sufficiently cue them during word retrieval difficulties. They mentioned that the robot could have asked additional questions and more in-depth questions to assist them formulating their responses.

Participant 11: *I can’t uh, not uh [participant moves hands alternately between himself and the robot], no uh, he’s not helping.*

Researcher: *He doesn’t help?*

Participant 11: *No.*

Researcher: *And how could he help? What could he do to make it easier?*

Participant 11: *Questions, something else, let’s say. How do you want that uh? Something else again ask so to say.* Researcher: *You mean he could repeat the question in a different way?*

Participant 11: *Yes, something like that. Another time. Deeper again. Still question.*

Researcher: *Some additional questions?*

Participant 11: *Yes yes.*

Researcher: *So, it would have been easier if he had asked more questions?*

Participant 11: *Yes, deeper so to say. That it uh that it, he helps with uh you can more uh questions answered so to say.*

Researcher: *Because then you could have told some more?*

Participant 11: *Yes yes.*

Participant 3: *Well, uh I uh I, now I have it again. Sometimes I can’t find the words and if that robot gives me a boost of uh, could it be this or could it be this, then he could help me, I think.*

#### Autonomy in Practising Language Use

The fourth theme that emerged was that participants (73%) indicated that they would like to increase the frequency in which they can practise language use in addition to language therapy (see Fig. 11). They emphasised the need for a conversation partner to practise language when they are alone. Practising language with a social robot was seen as a potential way to practise more frequent, tailored to individual needs in terms of frequency and practice duration.

Participant 6: *My uh I am furthermore uh good but uh, the first start of practise with aphasia then the robot was a uh added value in the sense of uh, then you can uh why do I say that, that exercises are limited in time. If you go to a rehabilitation centre, for example, you have 20 minutes of exercises with a speech therapist a day. Yes, that is limited. And that robot has all the time*.

Participant 6: *Well, uh, look uh, if you have aphasia you should be able to talk continuously because. Actually, you should also continuously have exercises*.

Participant 11: *You can practise. Practise, practise, practise. And if an alone here, all day. You cannot talk. And now [pp gestures to robot and herself] you can talk*.

Participant 3: *Well if no one is home, yes then you can uh without making an appointment, I think, have a small conversation with the robot*.

Apart from the three themes that emerged, it is also important to highlight that six participants (55%) used different kinds of nonverbal communication forms to express themselves. The nonverbal communication forms that participants used were (1) writing something down on a piece of paper or on a mobile device and then held it in front of their laptop camera; (2) gesturing; (3) gesturing accompanied by sound; and (4) showing an image on the telephone to the laptop camera. Participants used this augmentative communication when referring to the gestures the robot made, as well as when answering demographic questions about ‘year of birth’ and / or ‘highest degree of education’.

## Discussion

This study aimed to present a first-effort view on how PwA interact with the social robot NAO and how they perceived this interaction by observing, analysing, and assessing PwA’s interaction. In this section, findings are discussed along three factors underlying the success of self-administered technology-based treatment of PwA, as proposed by Macoir et al. [55]. These factors are: (1) technology-related factors—in this study the social robot NAO; (2) treatment-related factors—in this study the semi-structured interaction; and (3) user- related factors—in this study PwA.

### Technology-Related factors

The analyses suggest that participants did not perceive NAO as easy to use, which echoes in the theme *intelligibility of the robot* (55% perceived the gestures made by NAO as noisy), and in the observations regarding *communication break-down due to not hearing the robot properly*. In contrast, participants mentioned that NAO was *nice and easy to talk to*, had a *good speech rate*.

Based on these data, it appears that gestures made by NAO complicated the communication for participants instead of facilitating it. This contradicts previous studies, which demonstrated that PwA benefit from multimodal communication [30–34], because the use of congruent gestures by the conversation partner increases language comprehension [30]. The results from the current study suggest an opposite effect, namely that gestures made by NAO hindered the intelligibility and therefore may have also affected the comprehensibility of NAO. Participants indicated that the low intelligibility of NAO was caused by the noise of the motors of the robot while gesturing. This, in result, may have negatively affected participants’ perceived ease of use of NAO. At least in some cases, the low intelligibility of the NAO caused communication break-down, i.e., occurrences during interaction where participants did not seem to hear NAO properly. We suggest for future research to explore ways to overcome the disturbing noise due to the robot gesturing.

An alternative explanation for the perceived low intelligibility of NAO would be that the robot’s speech combined with gesturing increased the cognitive load of participants to such an extent that they experienced increased language comprehension problems. This is in line with the findings of Murray [56], who found a significant relationship between aphasia and attention deficits. So, it may be that participants did not actually experience intelligibility problems of NAO, but language comprehension problems as a result of attention problems. All in all, this may have negatively affected participants’ perceived ease of use.

Another possible explanation for the perceived low intelligibility of NAO as a result of gesturing would be that the interaction took place online. During video calling all sounds, i.e., the speech of the robot but also the sound of the robot’s motors while making gestures, were evenly amplified. As a result, the robot’s speech may have been perceived as less intelligible. This effect could have been reduced by advising participants to wear a headset, yet none of them did spontaneously. Alternatively, participants may have adjusted their audio settings after the initial question of the researcher whether they could hear the researcher properly. Unfortunately, the online setting made it infeasible to verify this repeatedly without disrupting the flow of the interaction. When examining the data of the *live participant* it is striking that this participant did perceive NAO as well intelligible, and in addition, did not encounter communication breakdowns.

Another factor that may have affected the *Perceived ease of use* is the *simultaneous turn-taking*, where the participant and the robot interrupted one another, or started talking at the same time. There are three possible explanations for these findings. First, the internet bandwidth of participants may have been too low causing the connection to hamper. This delay in signal reception may have caused participants to assume that the robot finished its turn, when in fact the robot was still taking its turn. This possible explanation, moreover, may explain why no simultaneously turn-taking was observed in the *live participant*, although this concerned only one participant. Second, comprehension problems of participants may have affected their turn-taking, i.e., if they did not understand or only partially understood what the robot said, they may not have expected the robot to continue its turn. Third, the researcher –who was a former aphasia therapist– acted as wizard during the interaction. She observed the nonverbal communication signals of participants indicating the process of word retrieval [43] before the robot took its turns. It may be, though, that the researcher missed some signals, since participants often fixated their gaze at the screen instead of the camera which interfered with the assessment of participants’ gaze (ibid.) Finally, the observational analysis indicated five occurrences of internet connection problems which may have negatively affected participants’ perceived ease of use.

Despite the aforementioned factors that may have negatively affected the *Perceived ease of use*, participants perceived NAO as *easy to talk to*. They indicated that NAO was nice and decent, although most participants were unable to explain in detail why they felt this way. This was most likely caused by cognitive problems, specifically problems in executive function skills, which may have complicated explaining their thought in more detail [56, 57]. Future research should acknowledge cognitive problems in PwA when using self-reported feedback. One possible explanation, though, for the fact that participants perceived NAO as *easy to talk to*, may be that (healthy) people who experience anxiety to talk to people, feel less anxiety when talking to a social robot compared to a human [58], however this was not tested on PwA. So, future research could assess whether this lowered anxiety effect also applies to PwA interacting with social robots. We, furthermore, suggest to investigate whether showing empathy by the robot through using emotional prosody in robot speech could facilitates communication.

Participants, furthermore, mentioned they appreciate the robot’s lowered speech rate, which may be an underlying factor that positively impacted their perception of NAO as *easy to talk to*. Moreover, the findings that participants denoted NAO as *easy to talk to*, are in line with Khosla et al. [27], who found that elderly interacted more intuitively with a social robot compared to an onscreen agent. Khosla et al. [27] demonstrated that social robots in elderly could assist overcome technological barriers. However, this was examined with elderly and the sample of the current study only contained one participant who can be regarded as such. So, future research could explore in more detail factors in PwA across age groups influencing their *Perceived ease of use* in interacting with social robots.

### Treatment-Related Factors

The analyses suggest that participants perceived NAO as useful, which emerges from the quantitative analysis of the questionnaire. Additionally, the theme *autonomy in practising language use*, brought to light how they thought the robot could be useful to them in practising language use.

Seventy-three percent of the participants would like to increase the frequency of practising language use, which might allow them to have a minimum of five to ten hours language training per week. This minimum is required to achieve improvement in language skills in everyday life [4, 5, 7]. Participants, furthermore, mentioned that they would like the treatment to be tailored to their specific needs, i.e., duration of the practise session [59]. We suggest that future research observes the interaction time to investigate possible effects on user performance.

Furthermore, 64% of the participants indicated that they were less satisfied with how the dialogue with NAO went during the interaction. They indicated that the robot did not seem to understand what they were saying, and that the robot, as a result, did not continue the conversation based on their responses. In other words, participants indicated that they would like the robot to adapt its behaviour in real-time based on their responses. As a result, the interaction would most likely mimic real live interaction, and would therefore be more useful for PwA to practise language. Cruz-Maya and Tapus [60] proposed a model, although not tested on PwA, in which the robot could adapt its behaviour based on the performance of the user and the user’s level of stress. In addition, Winkle et al. [28] concluded, in their study on user engagement based on interviews with rehabilitation therapists, that personalization of the interaction is essential for users to engage with the robot and maintain motivation. Similarly, van Minkelen et al. [29] found, although studied in preschool children, that personalization was the key element in maintaining motivation in interacting with the robot. It may be argued that the results regarding the perception of the dialogue in the current study are influenced by the effects of a semi-structured interaction where the researcher used WoZ to reflect an autonomous interaction. So future studies using Automatic Speech Recognition [41], as well as studies to test the model of Cruz-Maya and Tapus [60] in PwA seem necessary to overcome these limitations.

### User-Related Factors

The analyses suggest that participants enjoyed interacting with NAO, which is consistent with the literature showing the advantages of social robots over computer-based agents. For instance, Matarić et al. [35] found greater experienced joy in interacting with social robots compared to onscreen agents. Similarly, Wainer et al. [25], and Okamura et al. [24] found that people experienced the interaction with robots as more pleasant compared to onscreen agents.

Participants’ *Perceived Enjoyment* may have been negatively affected by the level of language comprehension problems participants experienced due to their aphasia. In other words, it may be that not fully understanding what the robot said resulting in a less enjoyable interaction with the robot.

The analyses, furthermore, revealed that participants felt positive toward using NAO. The construct *Attitude* refers to participants’ beliefs if NAO could be of practical use, i.e., the cognitive component of attitude [50]. This construct, however, might have been influenced by the voluntariness of all participants, since all participants felt positive about interacting with a social robot for this study.

The analyses, furthermore, suggest that participants specifically believed the robot could assist them practise language use independently of others, allowing them to personalise training frequency and duration [28, 55]. Almost all participants indicated that they needed to practise language use continuously to be able to improve their language skills. So, autonomy in practising language use seems an important factor in the actual use of technology [28, 55], in this study social robots, which also emerges from the theme *autonomy in practising language use*. PwA’s feeling of autonomy seem to be important factors in users’ engagement and motivation in using social robots [28, 29].

When examining participants’ engagement in the current study, which emerges from the observational category *Involvement* [45], most of the participants were properly involved, despite the occurrences of *communication break-dow*n and *simultaneously turn-taking* which are likely causes of lowered involvement. Winkle et al. [28] revealed that perceived enjoyment is an important factor for engagement. These findings seem to be in line with participants’ *Perceived enjoyment* in the current study which align with participants’ *Involvement*. It is possible that the level of participants’ engagement, is positively affected by the robot’s embodiment, in accordance with the findings by Okamura et al. [24]. They found that physical embodied robots yielded a more pleasant interaction, compared to onscreen agents, although one may argue that this effect is less strong in the current study due to the online design. The online design, in turn, may have affected participants’ *Mood*, i.e., the affective component of *Attitude* [50]. The analysis revealed that participants show a neutral mood, i.e., they showed no specific signs of liking or disliking NAO. Analyses of the current study showed no differences in *Mood* nor *Involvement* between the participants who interacted with NAO online vs. the participant who interacted with NAO live. So, future research could determine the effect and relevant factors of physical embodiment in a live setting vs. in an online setting on PwA’s engagement during an interaction with a social robot.

### Design Implications

Based on the results of this exploratory study, the following preliminary recommendations for robot designers emerge t in researching and developping social robots in providing additional language training to PwA.

#### Recommendation 1: Personalisation of the Interaction

The social robot should personalise its approach to the user by adapting its speech rate, volume, and pauses in real-time based on users’ language abilities and users’ responses. The robot should, in addition, detect when the user is no longer engaged and adapting its approach in real-time so that PwA’s autonomy in language practice remains preserved [28].

#### Recommendation 2: Interpretation of Alternative and Augmentative Communication Forms

PwA often use alternative and augmentative communication forms to support their language production, e.g., writing a single letter or word, drawing, showing a picture on a mobile device, or gesturing. Importantly, these forms of communication are influenced by the physical and cognitive constraints that PwA face because of their acquired brain injury. For example, a gesture is often made with one hand and drawing occurs with their non-preferred hand. As a result, these forms of communication are difficult to recognize but they nevertheless complement the spoken language production of PwA. Thus, for a social robot to provide language training to PwA, it should be able to recognize and adequately interpret various alternative and augmentative communication forms PwA could use to support their language production.

### Strengths and Limitations

The main strengths of this study are that (1) the results are based on a single online semi-structured interaction between 11 adults with mild to moderate chronic expressive aphasia and a NAO robot. To our knowledge, this is the first study to explore how PwA interact with a social robot; (2) the interaction as well as the questionnaire was designed by a researcher who was a former speech-language therapist and who has had almost 20 years of experience in working with PwA. This ensured the interaction as well as the questionnaire to be feasible and comprehensible to PwA; (3) the current study provides useful and novel evidence that PwA overall felt positive towards using a social robot.

There are limitations of this study that should be considered. First, given the small and specific sample of this study, generalisation should be treated with caution. We suggest a controlled follow-up study with a larger sample size to provide more conclusive findings. Since people with severe language comprehension problems (e.g., people with global aphasia, or people with Wernicke’s aphasia) were not included in this study, it is important to note than one should not draw conclusions beyond adults with mild to moderate chronic expressive aphasia. We suggest that future research will explore how people with other types of aphasia experience interacting with NAO.

Second, the qualitative component of the questionnaire may have been influenced by researcher bias, since the researcher used additional and clarifying questions to capture participants' opinion. However, the researcher recognized the pitfall of using leading questions beforehand and therefore always verified if the answer was indeed correctly interpreted. Moreover, the qualitative data should be interpreted with care since the robot was controlled using Wizard-fo Oz by the researcher who was a former aphasia therapist. The findings can therefore not be generalized across the population of people with mild to moderate chronic expressive aphasia.

Third, all participants voluntarily participated in this study, which may have affected their perception of the robot, although it is unlikely to assume that PwA will involuntarily use a robot for language training in the future.

Finally, the study took place online, except for one participant, due to the Covid-19 pandemic. As a result, participants could not experience a shared space with NAO, restraining the advantage of the physical presence of NAO. The findings of this exploratory study can therefore not be generalized to a real-live setting. A follow-up study in a real-live setting is therefore important to investigate how people with mild to moderate chronic expressive aphasia experience NAO.

So, it is desirable to conduct further research in real life with a larger sample of people with various types of aphasia to generalise the findings of this study. Moreover, this study should be replicated in real life, instead of online, to determine in more detail the relation between the robot’s gesturing and its intelligibility.

## Conclusion

This study contributed to the literature on deploying social robots to provide additional language training to PwA [7, 10], by providing a first step in exploring to what extent social robots can provide additional language training to adults with mild to moderate chronic expressive aphasia. A combination of quantitative and qualitative methods was used to explore how PwA interacted with the social robot NAO and how they perceived this interaction. The combined results from the observational analysis, thematic analysis and post-interaction questionnaire provides valuable design implications for social robots to meet the needs and requirements of PwA. The findings demonstrate that robots should personalise and adapt the interaction in real-time based on users’ responses, while maintaining user’s engagement. In addition, the robot should be able to recognize and respond to nonverbal communication forms used by PwA.

The findings of this study provide initial recommendations for the research and development of social robots for people with mild to moderate chronic expressive aphasia. These recommendations aim to enable PwA to practise language use independently of others by using a social robot, to increase practice intensity and frequency. This enables them to meet the required short-term high-intensive dose of five to ten hours language practice per week to enable significant improvements in language skills [5, 7].

## Figures and Tables

**Fig. 12:**
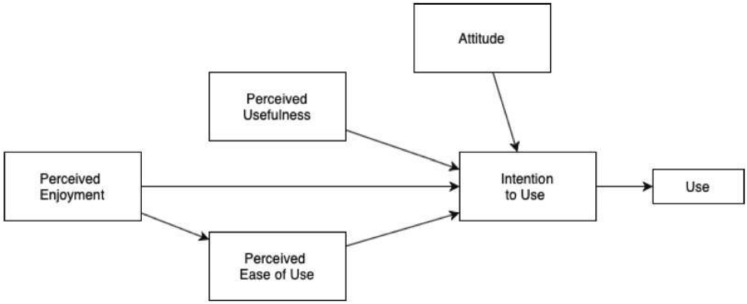
Model used to design the questionnaire, based on the Unified Theory of Acceptance and Use of Technology (UTAUT). (Venkatesh [49]) and the adaptation of Heerink et al. [48]

**Fig. 13:**
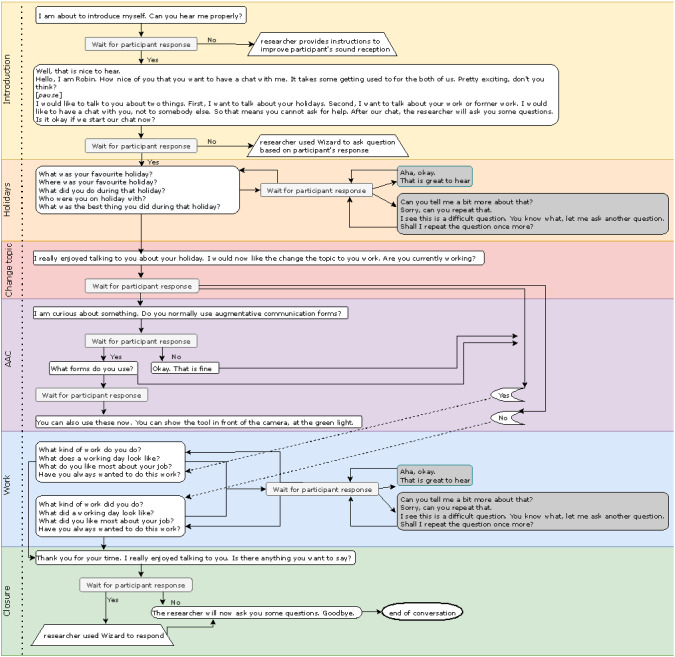
Flowchart showing the six phases of robot interaction

## Data Availability

The data of this study are available from the corresponding author (PvM) upon reasonable request.

## Appendix A

See Table 4.

**Table 4:** Coding scheme for interaction analysis between social robot NAO and people with aphasia

General categories	Subcategories
Simultaneously turn-taking	participant interferes robot’s speech robot interferes participant speech participant and robot start talking at the same time
Communication break-down	participant did not seem to understand the robot participant did not seem to hear the robot properly disturbing noise internet connection problems malfunction of the robot
Mood (*)	very happy/ excited happy/ satisfied neutral small signs of negative mood proper signs of negative mood very sad/ very negative mood
Involvement (*)	very engrossed/ involved properly involved neutral withdrawn

Categories with an asterisk are designed by Huisman and Kort [45]

## Appendix B

See Fig. 12.

## Appendix C

### Questionnaire items in Dutch

See Table 5.

**Table 5:** Questionnaire items of four constructs and demographics in Dutch

Questions	Construct	Mean	SD
1. Ik vind het leuk dat de robot met me praat	PE	3.73	1.01
2. Ik vind de robot aardig	PE	4.09	1.22
3. Ik vind de robot makkelijk om mee te praten	PEOU	3.00	1.00
4. Ik kan de robot goed verstaan	PEOU	3.73	0.91
5. Ik denk dat de robot handig is om taal mee te oefenen	PU	3.91	1.22
6. Ik zou graag meer gesprekken hebben met de robot	PU	3.64	1.21
7. Ik denk dat de robot een goede gesprekspartner kan zijn	ATT	3.73	1.01
8. Ik denk dat de robot mij kan helpen meer te oefenen met taal	ATT	3.82	1.33
9. Heeft u al eerder meegedaan aan een onderzoek met een sociale robot?	*NA*	*NA*	*NA*
10. Heeft u ooit eerder een gesprek gehad met een robot?	*NA*	*NA*	*NA*
11. Hoe omschrijft u uw gender?	*NA*	*NA*	*NA*
12. Wat is uw geboortejaar?	*NA*	52.09	13.61
13. Wat is de hoogste opleiding die u heeft afgerond?	*NA*	*NA*	*NA*

*PE* Perceived Enjoyment, *PEOU* Perceived Ease of Use, *PU* Perceived Usefulness, *ATT* Attitude, *DE* Demographic questions, *NA* not applicable

## Appenix D

See Fig. 13.

## Footnotes

1 https://www.softbankrobotics.com

2 http://doc.aldebaran.com/2-8/naoqi/motion/alanimationplayer-advanced.html

3 https://osf.io/qfkn5/
